# The Christmas Choirs at Cardiff

**Published:** 1913-01-04

**Authors:** 


### CHRISTMAS IN THE HOSPITALS.

### THE CHRISTMAS CHOIRS AT CARDIFF.

### Keeping the Octave at King Edward VII.'s Hospital.

Although there was nothing unusual in the observance of Christmas at King Edward VII.'s Hospital, Cardiff, the entertainment and good cheer provided were thoroughly enjoyed by everyone. In accordance with custom, the festivities commenced at five o'clock on Christmas Eve, when the gifts from the Christmas tree were distributed. The tree is placed each year in the children's ward, and as the children are at present occupying the new "Coronation" Ward, the scene was one of peculiar beauty, for the tile pictures illustrative of the Coronation and the Investiture of the Prince of Wales made a splendid background to the traditional tree, which blazed with electric lights and many coloured gifts. Still, it is doubtful if the children's engagement on Christmas Eve was more thorough than their happy interest in the previous days' preparation, in which they co-operated with the sister and nurses of the ward. Dr. Hairon, one of the house surgeons, made an admirable Father Christmas. He himself distributed probably over a hundred gifts, and accompanied each with an appropriate comment, the point of which was often appreciated not by the recipient alone. The Christmas tree was followed by an excellent concert in the "Jacob" Ward, contributed by the St. Saviour's Pierrots. In addition to the usual services on Christmas Day, each ward was visited during the afternoon by St. Peter's Choir, who delighted the patients by their singing. The sumptuous dinners on Christmas Day were quite obviously appreciated, the pleasure of the patients being greatly increased by the presence of members of the honorary and resident medical staff, who did the carving in their respective wards, with a liberal consideration for the patients' requirements. Boxing Day was relatively uneventful at the hospital, but on Friday evening the annual nurses' dance took place in the Out-patients' Hall. The entertainments will be continued during the present week with nightly concerts in one or other of the wards.

